# Selective fungal bioprecipitation of cobalt and nickel for multiple‐product metal recovery

**DOI:** 10.1111/1751-7915.13843

**Published:** 2021-06-11

**Authors:** John Ferrier, Laszlo Csetenyi, Geoffrey Michael Gadd

**Affiliations:** ^1^ Geomicrobiology Group School of Life Sciences University of Dundee Dundee DD1 5EH UK; ^2^ School of Science and Engineering Fulton Building University of Dundee Dundee DD1 5HN UK; ^3^ State Key Laboratory of Heavy Oil Processing Beijing Key Laboratory of Oil and Gas Pollution Control College of Chemical Engineering and Environment China University of Petroleum 18 Fuxue Road, Changping District Beijing 102249 China

## Abstract

There are a need for novel, economical and efficient metal processing technologies to improve critical metal sustainability, particularly for cobalt and nickel which have extensive applications in low‐carbon energy technologies. Fungal metal biorecovery processes show potential in this regard and the products of recovery are also industrially significant. Here we present a basis for selective biorecovery of Co and Ni oxalates and phosphates using reactive spent *Aspergillus* *niger* culture filtrate containing mycogenic oxalate and phosphate solubilized from struvite. Selective precipitation of oxalates was achieved by adjusting phosphate‐laden filtrates to pH 2.5 prior to precipitation. Co recovery at pH 2.5 was high with a maximum of ~96% achieved, while ~60% Ni recovery was achieved, yielding microscale polyhedral biominerals. Co and Ni phosphates were precipitated at pH 7.5, following prior oxalate removal, resulting in near‐total Co recovery (>99%), while Ni phosphate yields were also high with a recovery maximum of 83.0%.

## Graphical abstract

We provide a proof‐of‐concept demonstration of a versatile and selective Co and Ni biorecovery system based on fungal oxalate excretion and phosphate solubilization from struvite which provided a reactive culture filtrate capable of precipitating supplied Co or Ni as insoluble oxalates or phosphates.

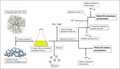

## Introduction

In recent years, much attention has focussed on improving the security of supply and sustainability of critical metal resources through the development of cheaper and environmentally friendly systems for metal processing and extraction from low‐grade ores, sludges and recycled materials (Watling, 2015; Werner *et al*., 2018). Co is one such critical metal resource that faces steep projected increases in demand because of strategically important industrial applications in alloys, electrochemical materials and catalysts (Petavratzi *et al*., 2019). Low‐cost metal processing technologies may improve the operational viability of Co extraction from under‐utilized resources, such as low‐grade ores and tailings, or those with additional costs associated with mining, e.g. deep‐sea manganese nodules and ferromanganese crusts.

Microbial alternatives or adjuncts to traditional hydrometallurgical technologies are now considered to be important options with regard to sustainable metal and mineral processing, with proven industrial applications over many years in the field of metal bioleaching (‘biomining’) (Watling, 2015; Johnson, 2018). Several other microbial processes for efficient biorecovery of metals from leachates and other metal‐laden solutions also show applied potential (Gallegos‐Garcia *et al*., 2008; Yang *et al*., 2020) and these rely on biomineralization and precipitation of metals from solution by direct or indirect processes involving microorganisms or their metabolites (Gadd and Pan, 2016; Yang *et al*., 2020).

In this work, we present a new system for selective, high‐yield biorecovery of cobalt (Co) and nickel (Ni), as their corresponding oxalates and phosphates. Ni is a valuable element that is frequently geochemically and industrially associated with Co, and therefore relevant to Co bioprocessing options. To achieve biorecovery of these two metals, we employed a reactive spent *Aspergillus* *niger* culture filtrate containing excreted oxalate and solubilized inorganic phosphate (P*_i_*) from the P‐containing mineral, struvite, which was incorporated in the medium. Since metal phosphates are soluble at low pH while simple divalent metal oxalates readily precipitate, we hypothesised that selective recovery of oxalates and phosphates could be achieved by manipulation of solution pH, thereby avoiding the simultaneous precipitation of oxalates and phosphates.

Oxalic acid secretion is central to a range of environmental processes mediated by fungi, including lignin degradation, plant pathogenesis, element cycling, and mineral colonization and bioweathering (Cessna *et al*., 2000; Fomina *et al*., 2010; Guggiari *et al*., 2011; Gadd *et al*., 2014; Ferrier *et al*., 2019). Furthermore, the ability of oxalate‐producing fungi, such as *A*. *niger*, to solubilize phosphate from insoluble inorganic sources is well known, including release from rock phosphate and other fertilizers as well as natural and synthetic struvite (Schneider *et al*., 2010; Mendes *et al*., 2015, 2013; Ceci *et al*., 2018; Suyamud *et al*., 2020). Such released mobile phosphate can subsequently precipitate with available metal species forming insoluble secondary metal phosphates, such as chloropyromorphite (Pb_5_(PO_4_)_3_Cl) with lead, and uranyl phosphates with uranium following depleted uranium or uranium oxide solubilisation by oxalate‐producing fungi (Fomina *et al*., 2007, 2008; Rhee *et al*., 2012. Struvite (NH_4_MgPO_4_·6H_2_O) precipitation has received considerable attention in the water industry for the recovery of phosphate from wastewaters and is produced commercially in several countries (Le Corre *et al*., 2007; Manning, 2008; Parsons and Smith, 2008; Peng *et al*., 2018. In water treatment plants, struvite can also extensively crystallize and accumulate in wastewater pipes leading to speculation as to the use of this waste product as a fertilizer (Le Corre *et al*., 2009). For these reasons, struvite was considered to be an appropriate source of inorganic phosphate for metal phosphate bioprecipitation, specifically the monohydrated form, dittmarite (NH_4_MgPO_4_·H_2_O). The objective of our work was to provide a proof‐of‐concept demonstration of a versatile and sustainable selective Co and Ni biorecovery system based on fungal oxalate excretion and phosphate solubilisation from struvite which provided a reactive culture filtrate capable of precipitating supplied Co or Ni as insoluble oxalates or phosphates.

## Results

### Efficiency of metal biorecovery

Following 14 days incubation of *A*. *niger* in struvite‐containing culture media, oxalate and phosphate concentrations in the biomass‐free culture supernatant were 19.94 ± 0.26 mM and 59.41 ± 0.43 mM respectively. These solutions were used to examine the efficiency of metal bioprecipitation on the addition of Co or Ni sulphate solutions at different reaction pH values; pH 2.5 for the precipitation of oxalate, and pH 7.5 for the precipitation of phosphates. These precipitation experiments were carried out separately, with the addition of excess CaCl_2_ at pH 2.5 to precipitate and extract oxalate, prior to pH adjustment to pH 7.5 and phosphate precipitation.

**Fig. 1:**
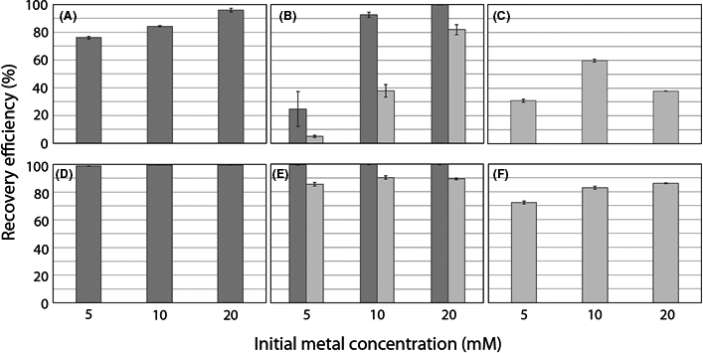
Co and Ni biorecovery using spent *A*. *niger* culture filtrate containing solubilized phosphate from struvite. Average Co and Ni biorecovery efficiencies are expressed as a percentage of the metal initially added that was removed from the solution as a precipitate. Charts show (A, D) Co recovery, (B, E) Co and Ni recovery from the mixed solutions and (C, F) Ni recovery. Dark bars represent Co, lighter bars represent Ni. Charts (A, B, C) show biorecovery efficiencies associated with precipitation at pH 2.5 for precipitation of oxalates, charts (D, E, F) show biorecovery efficiencies associated with precipitation at pH 7.5 following oxalate removal for the precipitation of phosphates. Metal concentrations were determined by AAS analysis using an AAnalyst 400 AA spectrophotometer and appropriate lamps and standard solutions. Samples and standard solutions were prepared using 1% HNO_3_. Error bars show ± 1 SD, *n* = 3.

At pH 2.5, the efficiency of Co biorecovery increased with increasing initial Co concentration with 76.1%, 84.3% and 95.9% being recovered from 5, 10 and 20 mM Co respectively (Fig. 1A). This also occurred for the mixed Co+Ni solutions, with 24.8% (5 mM Co+Ni), 92.6% (10 mM Co+Ni) and 99.9% (20 mM Co+Ni) Co being recovered (Fig. 1B). Ni recovery also increased with increasing Ni concentration in the mixed solutions, with 5.1% (5 mM Co+Ni), 37.9% (10 mM Co+Ni) and 82.0% (20 mM Co+Ni) recovery being achieved (Fig. 1B). Recovery from Ni solutions at pH 2.5 was 30.8% (5 mM Ni), 59.6% (10 mM Ni) and 37.9% (20 mM Ni) (Fig. 1C). At pH 7.5, following Ca‐oxalate precipitation, almost total Co recovery was achieved at every concentration tested with 99.8%, 99.4% and 99.5% recovery at 5, 10 and 20 mM initial Co concentrations respectively (Fig. 1D). Recovery of Co from the mixed Co+Ni solutions was 99.4%, 99.7% and 99.6%, at 5, 10 and 20 mM initial concentrations (Fig. 1E), while Ni recovery was 85.5%, 90.2% and 89.3% at 5, 10 and 20 mM initial concentrations respectively (Fig. 1E). Recovery of Ni from the single‐metal solution was 72.2%, 82.8% and 86.1% at 5, 10 and 20 mM initial Ni concentrations respectively (Fig. 1F).

### Characterization of metal precipitates

**Fig. 2:**
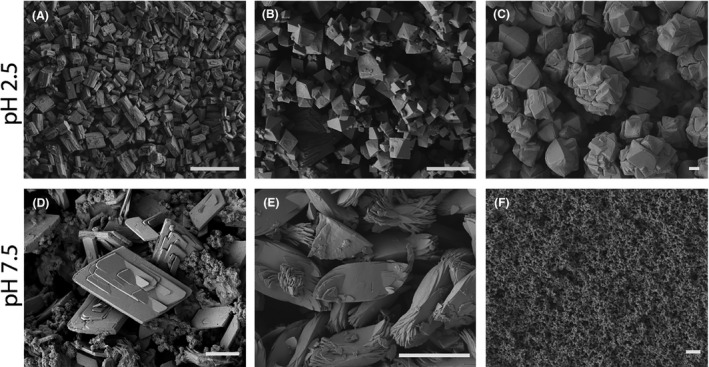
Scanning electron microscopy of precipitate morphologies. SEM images showing Co, Co+Ni and Ni precipitate morphology following precipitation using reactive spent culture filtrate adjusted to pH 2.5 or pH 7.5. Images are (A, B, C) Co, Co+Ni and Ni precipitates obtained at pH 2.5 from a 20 mM initial metal concentration respectively, (D) Co precipitates obtained at pH 7.5 from a 5 mM initial concentration, (E, F) Co, Co+Ni and Ni precipitates obtained at pH 7.5 from a 20 mM initial metal concentration respectively. Scale bars represent (A) 100 µm, (B, C, D, E) 10 µm and (F) 1 µm. Representative images are shown which were obtained using a JEOL SM‐7400F field emission scanning electron microscope at an accelerating voltage of 5 keV.

The Co precipitates generated at pH 2.5 formed orthorhombic micropolyhedral structures at each concentration tested (Fig. 2A). The mixed Co+Ni precipitates typically formed rhombohedral micropolyhedrons at pH 2.5 (Fig. 2B), while the Ni precipitates formed hexagonal microscale polyhedrons with frequent twinning (Fig. 2C). At pH 7.5, the Co precipitates formed monoclinic micropolyhedral structures across all concentrations tested (Fig. 2D), with nanoplate structures also being observed. The Co+Ni precipitates formed flat elliptical particles (Fig. 2E), while the Ni precipitates formed amorphous nanoparticles across all concentrations tested (Fig. 2F). Further scanning electron microscopy (SEM) images of the biominerals precipitated at each pH and metal concentration tested are shown in the Supplementary Information (Figs S1 and S2).

**Fig. 3:**
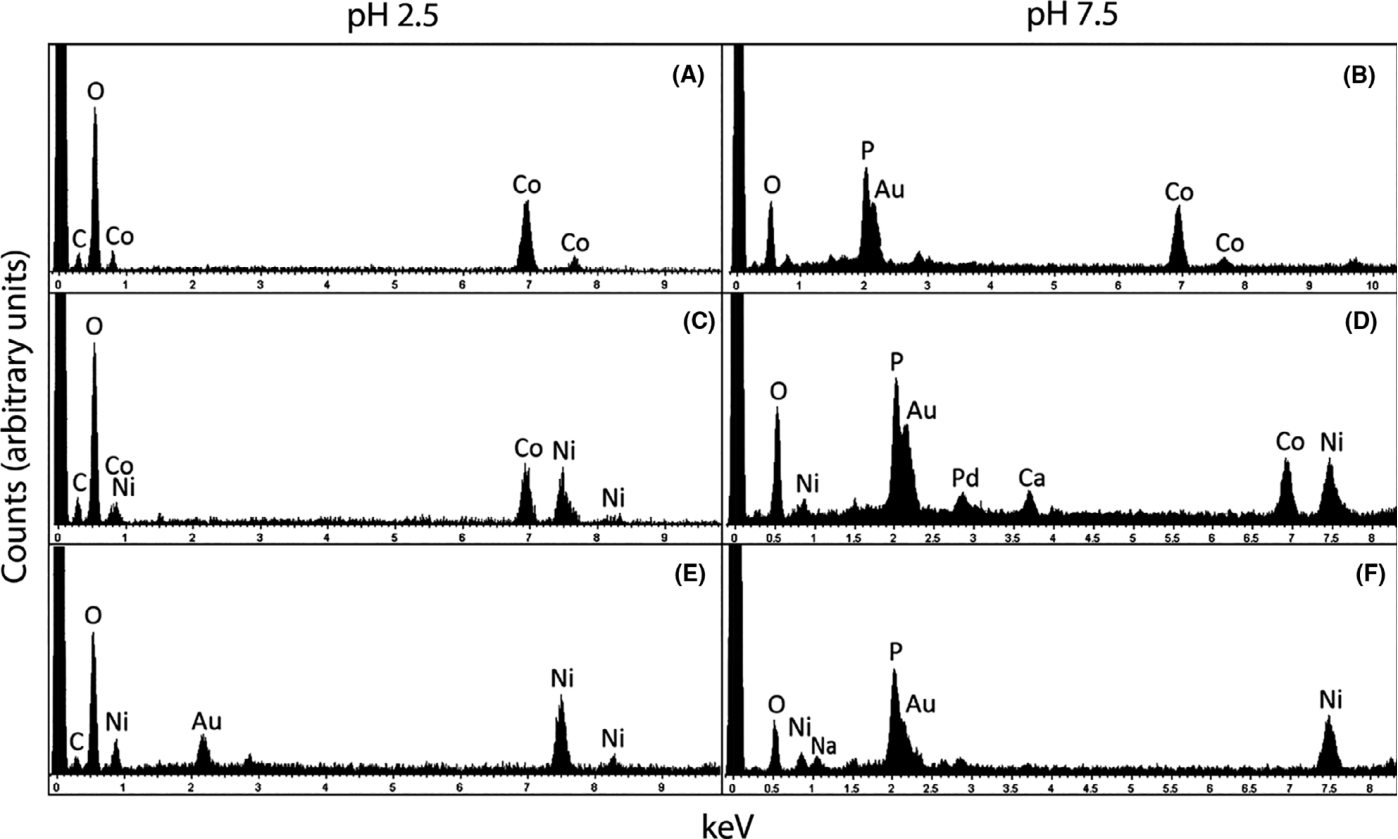
Elemental composition of precipitates. Typical EDXA spectra obtained from (A) Co precipitates, (C) Co+Ni oxalate precipitates and (E) Ni oxalate precipitates obtained using biomass‐free *A*. *niger* struvite culture media adjusted to pH 2.5, (B) Co precipitates, (D) Co+Ni oxalate precipitates and (F) Ni oxalate precipitates obtained using biomass‐free *A*. *niger* struvite culture media adjusted to pH 7.5. Analysis was carried out using a JEOL SM‐7400F field emission scanning electron microscope at an accelerating voltage of 20 keV.

The elemental composition of the precipitates was determined by energy dispersive X‐ray analysis (EDXA) (Fig. 3). At pH 2.5, the Co precipitates showed peaks for C, O and Co (Fig. 3A), the mixed Co+Ni precipitates showed peaks for C, O, Co and Ni (Fig. 3C), while the Ni precipitates showed peaks for C, O, and Ni, the Au peak arising from the sample coating (Fig. 3E). In contrast, at pH 7.5 the Co precipitates showed peaks for C, P and Co (Fig. 3B), the Co+Ni precipitates showed peaks for O, Ni, P, Ca, Co and Ni (Fig. 3C), and the Ni precipitates showed peaks for O, Ni, Na and P (Fig. 3F). There was therefore a clear difference in the elemental composition of the precipitates obtained at the two pH values, notably the presence of P only in that obtained at pH 7.5.

**Fig. 4:**
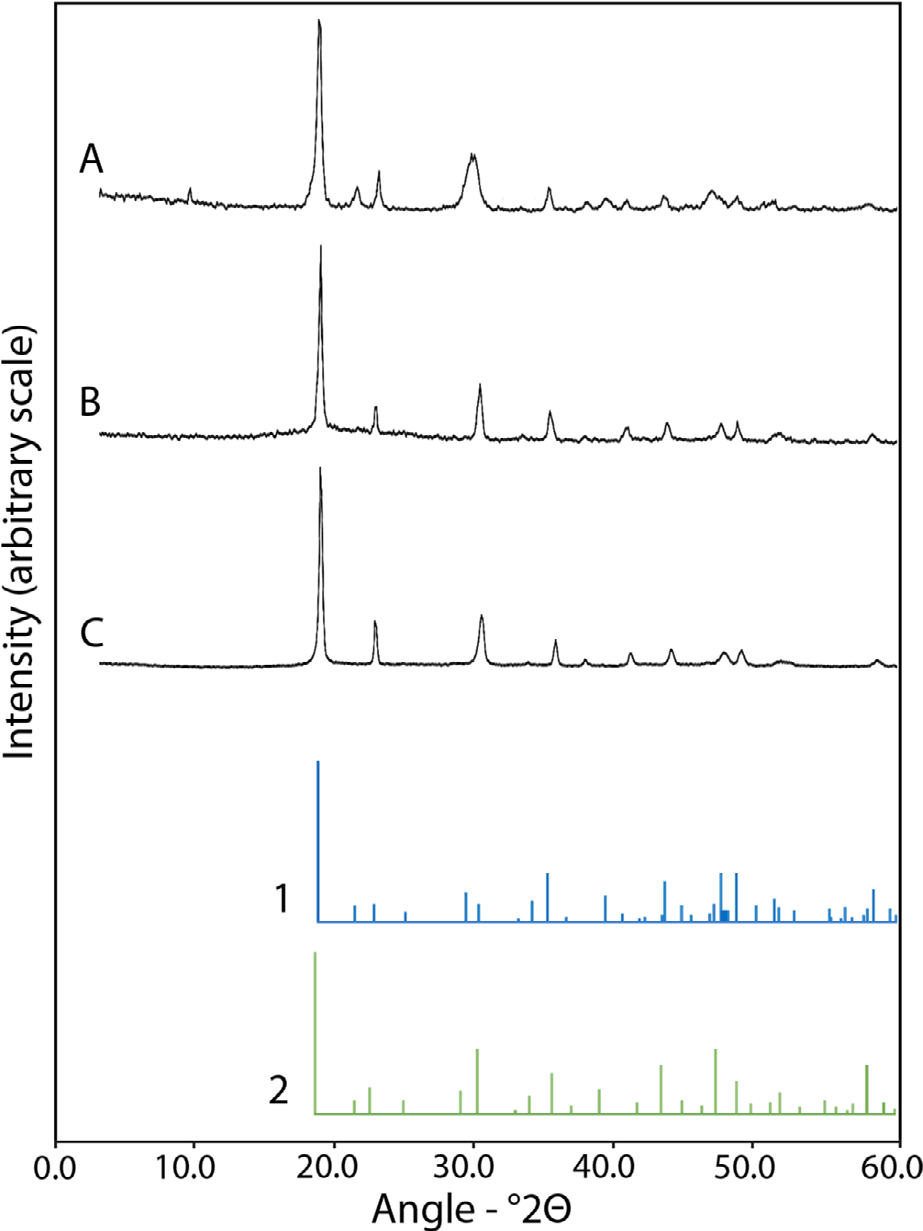
Mineralogical composition of oxalate precipitates. Typical XRD patterns obtained from (A) Co, (B) Co+Ni and (C) Ni oxalate precipitates obtained using biomass‐free *A*. *niger* struvite culture media adjusted to pH 2.5. Reference patterns correspond to (1) cobalt oxalate dihydrate (CoC_2_O_4_∙2H_2_O, 25‐251) and (2) nickel oxalate dihydrate (NiC_2_O_4_∙2H_2_O, 25‐582) and were obtained from the IDCC database. Sample replicates were pooled and subsamples analysed using a Siemens D5000 powder X‐ray diffractometer.

X‐ray diffraction (XRD) patterns obtained for the pH 2.5 precipitates at a 20 mM initial metal concentration showed that the Co precipitates were Co oxalate dihydrate (CoC_2_O_4_∙2H_2_O) (Fig. 4A) while the Ni precipitates were Ni oxalate dihydrate (NiC_2_O_4_∙2H_2_O) (Fig. 4C). Although the XRD patterns were very similar, it was concluded that the mixed (Co+Ni) precipitates contained both CoC_2_O_4_∙2H_2_O and NiC_2_O_4_∙2H_2_O (Fig. 4B), especially when considering the elemental composition of the mixed precipitates (Fig. 3C).

**Fig. 5:**
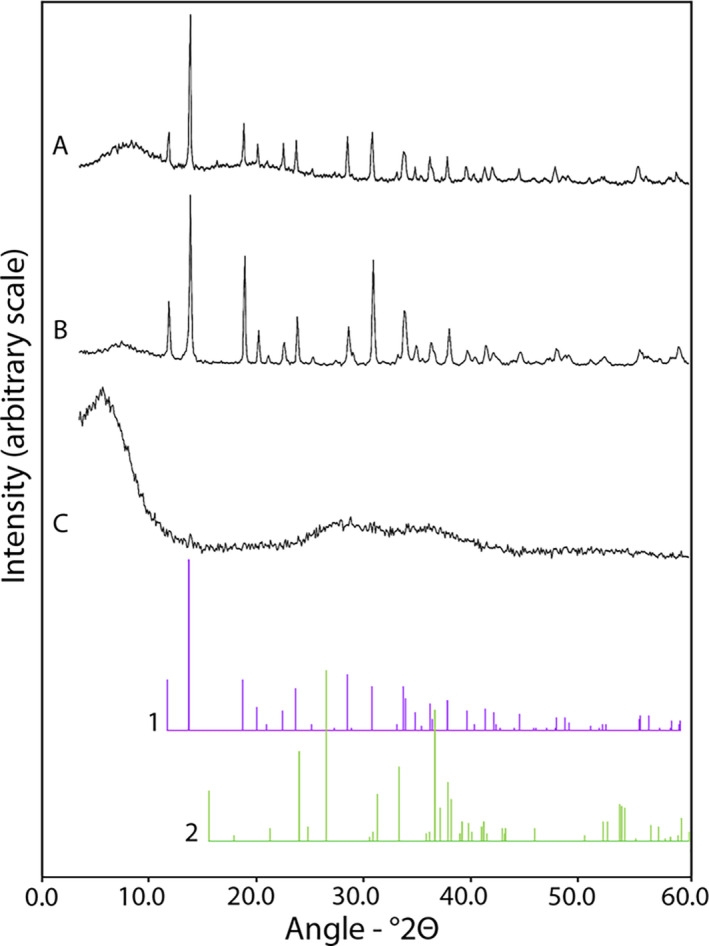
Mineralogical composition of phosphate precipitates. Typical XRD patterns obtained from (A) Co, (B) Co+Ni and (C) Ni precipitates obtained using biomass‐free *A*. *niger* struvite culture media adjusted to pH 7.5 for phosphate precipitation after an initial oxalate removal stage. Reference patterns correspond to (1) cobalt phosphate octahydrate (Co_3_(PO_4_)_2_∙8H_2_O, 41‐375), (2) nickel phosphate (Ni_3_(PO_4_)_2_, 38‐1473) and were obtained from the IDCC database. Sample replicates were pooled and subsamples analysed using a Siemens D5000 powder X‐ray diffractometer.

The Co and Co+Ni precipitates obtained at pH 7.5 (Fig. 5Aand B) generated XRD patterns corresponding to Co phosphate octahydrate (Co_3_(PO_4_)_2_∙8H_2_O). The Co+Ni precipitates showed some XRD evidence for Ni phosphate octahydrate (Ni_3_(PO_4_)_2_∙8H_2_O), although this was inconclusive. The XRD pattern for the Ni precipitates indicated an amorphous nature, showing a broad peak roughly corresponding to major reference peaks at around 26° and 36° (Fig. 5C). The amorphous nature of the Ni precipitates may explain the lack of clear Ni phosphate XRD patterns for the mixed Co+Ni precipitates. These XRD patterns closely match published XRD data for Co and Ni phosphates (Tang *et al*., 2016), and cumulatively, our data confirm that the pH 7.5 precipitates were Co, Co, Ni and Ni phosphates.

## Discussion

A shift towards new sustainable concepts in metal processing industries has led to the investigation of bioprecipitation approaches for the recovery of soluble metal species from solution, notwithstanding the long‐standing appeal of such systems for the bioremediation of metal‐contaminated wastewaters (Cessna *et al*., 2000; Gadd and Pan, 2016; Liang and Gadd, 2017). In this work, XRD and EDXA analysis confirmed that oxalates were selectively precipitated from solution at pH 2.5. Particularly high Co oxalate precipitation was achieved for both single and mixed‐metal solutions, which contrasted with Ni showing consistently lower recovery efficiencies relative to Co. This can be explained by the lower solubility product of CoC_2_O_4_∙2H_2_O (*K*_sp_ = 2.7 × 10^−9^) compared with NiC_2_O_4_∙2H_2_O (*K*_sp_ = 1.2 × 10^−3^) (Li *et al*., 2014). With the exception of Ni alone, the highest recoveries were obtained at a 20 mM initial metal concentration, and while Ni recovery at 20 mM was less than at a 10 mM concentration in terms of efficiency, in absolute terms a greater amount of Ni oxalate was precipitated at a 20 mM concentration. While oxalates of divalent metals are sparingly soluble or insoluble and readily precipitate from solutions, soluble metal complexes can also form, comprised of oxalate anions linked to a central metal cation via coordinate bonds (Verma *et al*., 2019). The frequency of oxalate complex formation increases with excess oxalate and this may contribute to the reduced Co and Ni recovery efficiency at lower initial concentrations due to the lower metal ion to oxalate ratios since both Co^2+^ and Ni^2+^ can form M(C_2_O_4_)_2_^2−^ and M(C_2_O_4_)_3_^4−^ complexes (Krishnamurty and Harris, 1960; Tang *et al*., 2016). Following oxalate removal by Ca‐oxalate precipitation, phosphate precipitation at pH 7.5 was carried out. Phosphate precipitation showed very high recovery efficiencies, with near‐total Co being recovered at each concentration tested in both single and mixed‐metal solutions, while Ni recovery reached 86.1% in the single‐metal precipitations and over 85% in the mixed‐metal solutions at each concentration tested. As before, the dominance of Co recovery from the mixed‐metal solutions may be explained by the differing solubility products of Co_3_(PO_4_)_2_ (*K*_sp_ = 2.05 × 10^‐35^) and Ni_3_(PO_4_)_2_ (*K*_sp_ = 4.74 × 10^‐32^), although both phosphates are highly insoluble and Co recovery was only ~10–15% more efficient than that of Ni. XRD analysis showed clear patterns for Co phosphate in the single‐metal Co and mixed‐metal precipitates. Clear XRD patterns were not detected for Ni phosphate, and the XRD patterns obtained for the Ni precipitates only showed two broad peaks which broadly corresponded with the highest‐intensity reference peaks at 26° and 36°. The absence of clear Ni phosphate patterns in the mixed‐metal precipitates was primarily due to their amorphous nanoscale morphology. However, the clear peaks for P determined using EDXA provided supporting evidence that these precipitates were phosphates. Similar XRD patterns have been reported for Co, Co+Ni and amorphous Ni phosphates in other studies (Tang *et al*., 2016). Selective metal phosphate precipitation was attempted at pH 7.5 without oxalate removal, but this resulted in simultaneous precipitation of both oxalates and phosphates (data not shown). The data presented here show clearly that phosphates may be selectively precipitated from the solution following an oxalate removal step. Future investigations could seek to optimise a sequential approach, involving total oxalate removal as Co/Ni oxalates, prior to phosphate precipitation.

**Fig. 6:**
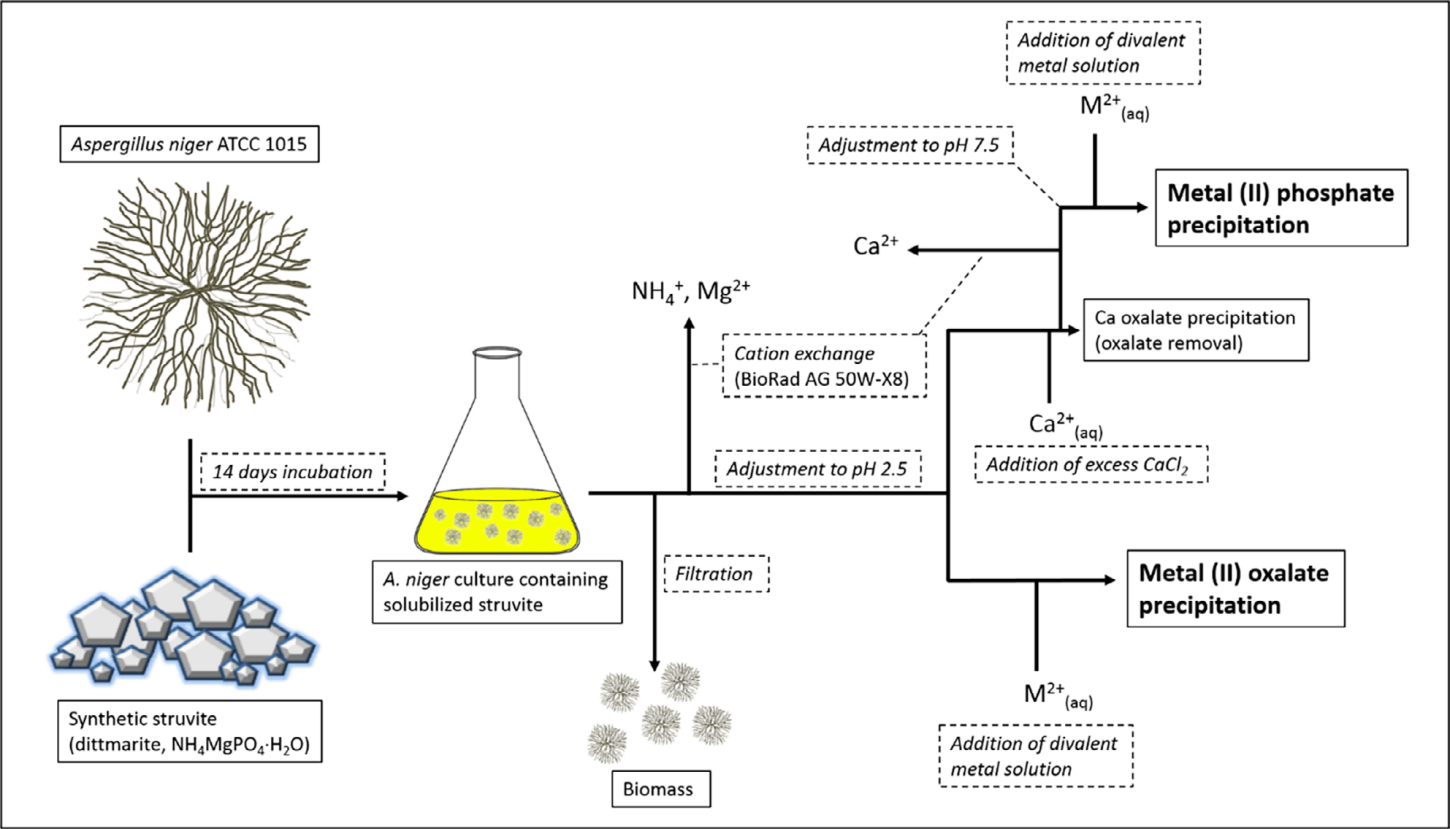
Overview of the *A*. *niger* struvite reactive spent culture filtrate, metal oxalate and phosphate biorecovery system. The diagram demonstrates the stages required to prepare the reactive oxalate‐ and phosphate‐laden supernatant, following appropriate pH adjustment required for final product precipitation. Protocol stages are displayed in dashed boxes with text in italics, inputs and products are shown in solid boxes with standard text.

A simplified process summary is shown in Fig. 6. In this work, oxalate was removed by the addition of an excess of CaCl_2_ resulting in calcium oxalate precipitation, which clearly facilitates the efficient biorecovery of pure metal phosphates. Our proposed metal biorecovery system may be envisioned as a modular process that could be applied in combination with any metal processing technologies which produce an aqueous metal‐containing solution, such as wastewaters and streams, hydrometallurgical processing streams and leachates, and recycling processes. While this study used struvite due to its established use in phosphate recovery from wastewaters, and also availability as waste material from water treatment facilities, both aspects implicit with resource sustainability, there is scope for the use of other inorganic phosphate‐containing materials that can be solubilized, such as tricalcium phosphate (TCP), bone char, and rock phosphate (Mendes *et al*., 2015, 2013; Ceci *et al*., 2018; Suyamud *et al*., 2020). Co and Ni oxalates and phosphates are industrially significant materials and receive wide‐ranging applications as catalysts, pigments and, in the case of Co/Ni oxalates, can be used as precursor materials for the production of oxides which are components of lithium‐ion batteries and other energy storage devices (Wang *et al*., 2011; Che *et al*., 2013; Kim *et al*., 2015; Sun *et al*., 2020). Co and Ni oxalates and phosphates are also receiving attention for direct application as energy storage materials and appear particularly suited for application in batteries and supercapacitors (Zhang *et al*., 2015; Li and Gadd, 2017; Theerthagiri *et al*., 2017; Li *et al*., 2018; Yeoh *et al*., 2018). There is, therefore, a clear opportunity to explore the suitability of fungal biorecovery products for useful future applications in this area. In summary, a fungal‐struvite biorecovery system showed potential as a low‐cost high‐yield Co and Ni biorecovery system through the production of micro‐ and nanoscale biomineral products. **I**n principle, such a process could be applied to any metals which form insoluble metal oxalates and phosphates.

## Experimental procedures

### Organism, media and growth conditions

*Aspergillus niger* 1015 was maintained on 90 mm diameter malt extract agar (MEA) plates (LabM, Bury, UK) at 25 °C in the dark. Liquid AP1 medium of the following composition was used (mmol l^−1^ Milli‐Q water): 111.01 D‐glucose, 37.86 (NH_4_)_2_SO_4_, 36.74 KH_2_PO_4_, 0.81 MgSO_4_∙7H_2_O, 0.23 CaCl_2_∙6H_2_O, 1.71 NaCl (Sigma‐Aldrich Merck, Irvine, UK), 9.25 × 10^−3^ FeCl_3_∙6H_2_O. The following trace metals were also included (mmol l^−1^ Milli‐Q water): 1.39 × 10^−2^ ZnSO_4_∙7H_2_O, 1.79 × 10^−2^ MnSO_4_∙4H_2_O and 1.6 × 10^−3^ CuSO_4_∙5H_2_O. Media components were autoclaved separately, before combining to the concentrations stated and distributing 300 ml portions into sterile 500 ml Erlenmeyer flasks. Flasks were amended with sterile synthetic struvite to a concentration of 1% (w/v) and inoculated using an *A*. *niger* spore suspension, prepared in 0.2% Tween 80, to an initial concentration of 5 × 10^5^ spores ml^−1^. *A*. *niger* spore suspension was prepared by adding 20 ml sterile 0.2% Tween 80 to *A*. *niger* slants prepared on MEA, which were then shaken vigorously to suspend the spores. The resultant suspension was then filtered aseptically through miracloth (Millipore, Livingston, UK) and the spore concentration determined by counting using a Neubauer chamber, before diluting to 5 × 10^7^ spores ml^−1^ using Milli‐Q water. Flasks were incubated for 14 days at 25 °C with shaking at 125 rpm in the dark. Following incubation, the biomass was removed by filtering through 11.0 cm diameter filter papers (Whatman plc, GE Healthcare, Little Chalfont, UK). The reactive spent culture filtrate was further vacuum filtered through 0.45 µm pore size, 47 mm diameter cellulose nitrate membrane filters (Whatman plc, GE Healthcare, Little Chalfont, UK).

### Struvite preparation

Synthetic struvite (dittmarite) was prepared using a solution containing (mmol l^−1^ Milli‐Q water): 31.91 MgCl_2_ (Merck Millipore, Watford, UK), 101.1 NH_4_Cl (BDH Chemicals, Poole, UK), 44.36 K_2_HPO_4_ (VWR International, Lutterworth, UK). This solution was adjusted to pH 8.5 using 5 M NaOH (VWR International, Lutterworth, UK) and stirred continuously for 10 min. The precipitate was then recovered by centrifugation (101 *g* × 15 min), with the supernatant being discarded, replaced with Milli‐Q water and the struvite resuspended. Centrifugation and washing with Milli‐Q water were repeated twice. The struvite was dried in a desiccator to constant weight and confirmed as dittmarite (NH_4_MgPO_4_·H_2_O) by X‐ray diffraction (XRD). The struvite was ground to a fine powder using a mortar and pestle prior to sterilization at 105 °C for at least 24 h prior to use.

### Precipitation of cobalt and nickel oxalates and phosphates

In this work, oxalate and phosphate precipitation procedures were conducted separately. Precipitation experiments for Co, mixed Co+Ni and Ni solutions (all sulphates) were conducted in triplicate at 5, 10 and 20 mM concentrations. Prior to use, *A*. *niger* struvite spent culture medium was treated with Biorad AG 50W‐X8 cation exchange resin (Bio‐Rad Laboratories Ltd., Watford, UK) to remove cations (Mg^2+^ and NH_4_^+^) and prevent reprecipitation of struvite during pH adjustment. Biorad AG 50W‐X8 cation exchange resin was added to the spent culture medium to a 10% (w/v) concentration and shaken for at least 2 h at 60 rpm, before filtering through 11.0 cm diameter filter paper. Following cation removal, the pH of the spent medium was pH 1.13. This cation‐free reactive spent culture filtrate was adjusted to pH 2.5 with 5 M NaOH for precipitation of oxalates or pH 7.5 for the precipitation of phosphates. Stock metal solutions were prepared using CoSO_4_∙7H_2_O and NiSO_4_∙6H_2_O at 50, 100 and 200 mM and the mixed Co+Ni stock solutions were prepared with Co:Ni in a 1:1 ratio. Precipitation was achieved by adding 1ml aliquots of stock Co, mixed Co+Ni and Ni solution to 9ml aliquots of cation‐free reactive spent culture filtrate to give final metal concentrations of 5, 10, 20 mM. Prior to phosphate precipitation, oxalate was removed by addition of excess CaCl_2_ (1ml 600 mM CaCl_2_ in 9ml filtrate to give a final concentration of 60 mM) resulting in Ca‐oxalate precipitation. This was removed by centrifugation (7280 *g* × 15 min) and the supernatant filtered through Sartorius Minisart 0.2 µm pore size cellulose nitrate syringe filters before being treated again with AG 50W‐X8 cation exchange resin as previous. All precipitations were conducted with mixing for 24 h on a rotary shaker at 60 rpm at room temperature. Precipitates were recovered by centrifugation (7280 *g* × 15 min) and washed twice by suspending in Milli‐Q water and repeating the centrifugation.

### Inorganic phosphate (P_i_) quantification

Measurement of P*_i_* concentrations in the reactive spent culture filtrate was determined using the molybdenum blue assay (Murphy and Riley, 1962). Three reagents were initially prepared: 2.5 M sulphuric acid (H_2_SO_4_), 32.33 mM ammonium molybdate ((NH_4_)_6_Mo_7_O_24_) and 8.21 mM potassium antimony tartrate (C_8_H_10_K_2_O_15_Sb_2_). With these reagents, a mixed solution was then prepared containing: 528 mg ascorbic acid (C_6_H_8_O_6_), 50 ml 2.5 M H_2_SO_4_, 15 ml 32.33 (NH_4_)_6_Mo_7_O_24_, 5 ml C_8_H_10_K_2_O_15_Sb_2_ and made up to 100ml with Milli‐Q water. P*_i_* standards were prepared from 0‐1 mg L^‐1^ P*_i_*, using a KH_2_PO_4_ stock solution, and samples were diluted in Milli‐Q water prior to analysis. 0.8 ml of the mixed solution was added to 5 ml aliquots of diluted reactive spent culture filtrate or P*_i_* standard solution and mixed for 10 min. 1ml aliquots of these solutions were then added to 3.5 ml polystyrene cuvettes (Sarstedt AG & Co, Hemer, Germany) and the absorbance at 882 nm measured using an Ultrospec 2100 pro UV/Visible Spectrophotometer (Amersham Biosciences, GE Healthcare, Little Chalfont, UK).

### pH measurements

Measurements of pH were taken using a HI‐1144B pH electrode with a pH 210 Microprocessor pH metre (Hanna Instruments, Leighton Buzzard, UK).

### Atomic absorption spectrophotometry (AAS)

Metal recovery efficiency was determined using an AAnalyst 400 AA Spectrometer (PerkinElmer Ltd, Beaconsfield, UK) using the appropriate lamps and standard solutions. All samples were filtered using Sartorius Minisart 0.2 µm pore size cellulose nitrate syringe filters (Sartorius Stedim UK Ltd., Epsom, UK) and diluted to an appropriate concentration using 1% HNO_3_ prior to analysis. Analyses were conducted in triplicate and reported concentrations are means of three individual readings. Data processing was conducted using Microsoft Excel (Microsoft Corporation, Reading, UK).

### High‐performance liquid chromatography (HPLC)

Final oxalate concentrations in the reactive spent culture filtrate were measured using a Dionex UltiMate 3000 HPLC system (Dionex Ltd., Camberley, UK) using appropriate standard solutions and a 5 mM H_2_SO_4_ mobile phase. Standards and samples were filtered using Sartorius Minisart 0.2 µm pore size cellulose nitrate syringe filters and the mobile phase was vacuum filtered through 0.45 µm cellulose nitrate membrane filters prior to analysis. A Biorad Fast Acid Analysis HPLC column #125‐0100 (Bio‐Rad Laboratories Ltd., Watford, UK) was used for analysis and data processing was conducted using the Chromeleon 6.8 data system. A flow rate of 1 ml min^‐1^ was used, with a 5 min sample run time and a nominal temperature of 40 °C.

### Scanning electron microscopy (SEM) and energy dispersive X‐ray analysis (EDXA)

Precipitates were mounted on 25 mm × 5 mm aluminium stubs using double‐sided adhesive carbon tape. The precipitates were suspended in small quantities of Milli‐Q water and pipetted onto the tape before drying in a desiccator for 2 days and coated with gold and palladium using a Cressington 208HR sputter coater (Cressington Scientific Instruments, Watford, UK). For coating, the mounted samples were added to the sputter coater vacuum chamber, the lid replaced and the pump initiated. Once a vacuum was achieved, Ar was leaked into the chamber and coating initiated and ceased upon reaching a 25 nm coat thickness. SEM and EDXA analysis was conducted using a field scanning electron microscope (JEOL SM‐7400F), operating at an accelerating voltage of 5 kV for imaging and 20 kV for EDXA.

### X‐ray diffraction (XRD)

XRD analysis was conducted using a Siemens D5000 powder X‐ray diffractometer (Siemens Healthineers, Henkestraße 127, 91052 Erlangen, Germany). Samples were ground to a fine powder using a mortar and pestle before being applied to PVC slides. Diffraction patterns were recorded using angular increments of 0.1° 2Θ from 3° to 60° 2Θ, at a rate of 1° 2Θ/min. A Cu‐Kα source was used, operating at 40 mA and 40 kV, with a scintillation detector. Reference patterns were obtained from the International Centre for Diffraction Data (IDCC) database.

## Conflict of interest

The authors declare no conflicts of interest.

## Supporting information


**Fig. S1**. Oxalate biomineral precipitate morphologies. SEM images showing Co, Co‐Ni and Ni precipitate morphology following precipitation using *A*. *niger* synthetic struvite supernatants adjusted to pH 2.5. Images are (A, B, C) Co, Co‐Ni and Ni precipitates obtained from a 5 mM initial metal concentration respectively, (D, E F) Co, Co‐Ni and Ni precipitates obtained from a 10 mM initial metal concentration respectively and (G, H, I) Co, Co‐Ni and Ni precipitates obtained from a 20 mM initial metal concentration respectively. Scale bars represent (A, G) 100 μm, (B, D, E, H) 10 μm and (C, F, I) 1 μm. Representative images are shown and were obtained using a JEOL SM‐7400F field emission scanning electron microscope operating at an accelerating voltage of 5 keV.
**Fig. S2**. Phosphate biomineral precipitate morphologies. SEM images showing Co, Co‐Ni and Ni precipitate morphology following precipitation using *A*. *niger* struvite supernatants adjusted to pH 7.5 for phosphate precipitation following oxalate removal. Images are (A, B, 26 C) Co, Co‐Ni and Ni precipitates obtained from a 5 mM initial metal concentration respectively, (D, E F) Co, Co‐Ni and Ni precipitates obtained from a 10 mM initial metal concentration respectively and (G, H, I) Co, Co‐Ni and Ni precipitates obtained from a 20 mM initial metal concentration respectively. Scale bars represent 10 μm and (C, I) 1 μm. Representative images are shown and were obtained using a JEOL SM‐7400F field emission scanning electron microscope operating at an accelerating voltage of 5 keV.
