# Supplementary material for: Selective fungal bioprecipitation of cobalt and nickel for multiple‐product metal recovery
Source: Microb Biotechnol. 2021 Jun 11;14(4):1747–56. doi: 10.1111/1751-7915.13843 (PMC8313247; doi:10.1111/1751-7915.13843)
Supplement: Supplementary file 1 — Fig. S1. Oxalate biomineral precipitate morphologies. SEM images showing Co, Co‐Ni and Ni precipitate morphology following precipitation using A. niger synthetic struvite supernatants adjusted to pH 2.5. Images are (A, B, C) Co, Co‐Ni and Ni precipitates obtained from a 5 mM initial metal concentration respectively, (D, E F) Co, Co‐Ni and Ni precipitates obtained from a 10 mM initial metal concentration respectively and (G, H, I) Co, Co‐Ni and Ni precipitates obtained from a 20 mM initial metal concentration respectively. Scale bars represent (A, G) 100 μm, (B, D, E, H) 10 μm and (C, F, I) 1 μm. Representative images are shown and were obtained using a JEOL SM‐7400F field emission scanning electron microscope operating at an accelerating voltage of 5 keV. Fig. S2. Phosphate biomineral precipitate morphologies. SEM images showing Co, Co‐Ni and Ni precipitate morphology following precipitation using A. niger struvite supernatants adjusted to pH 7.5 for phosphate precipitation following oxalate removal. Images are (A, B, 26 C) Co, Co‐Ni and Ni precipitates obtained from a 5 mM initial metal concentration respectively, (D, E F) Co, Co‐Ni and Ni precipitates obtained from a 10 mM initial metal concentration respectively and (G, H, I) Co, Co‐Ni and Ni precipitates obtained from a 20 mM initial metal concentration respectively. Scale bars represent 10 μm and (C, I) 1 μm. Representative images are shown and were obtained using a JEOL SM‐7400F field emission scanning electron microscope operating at an accelerating voltage of 5 keV. [file MBT2-14-1747-s001.pdf]

Supplementary Information

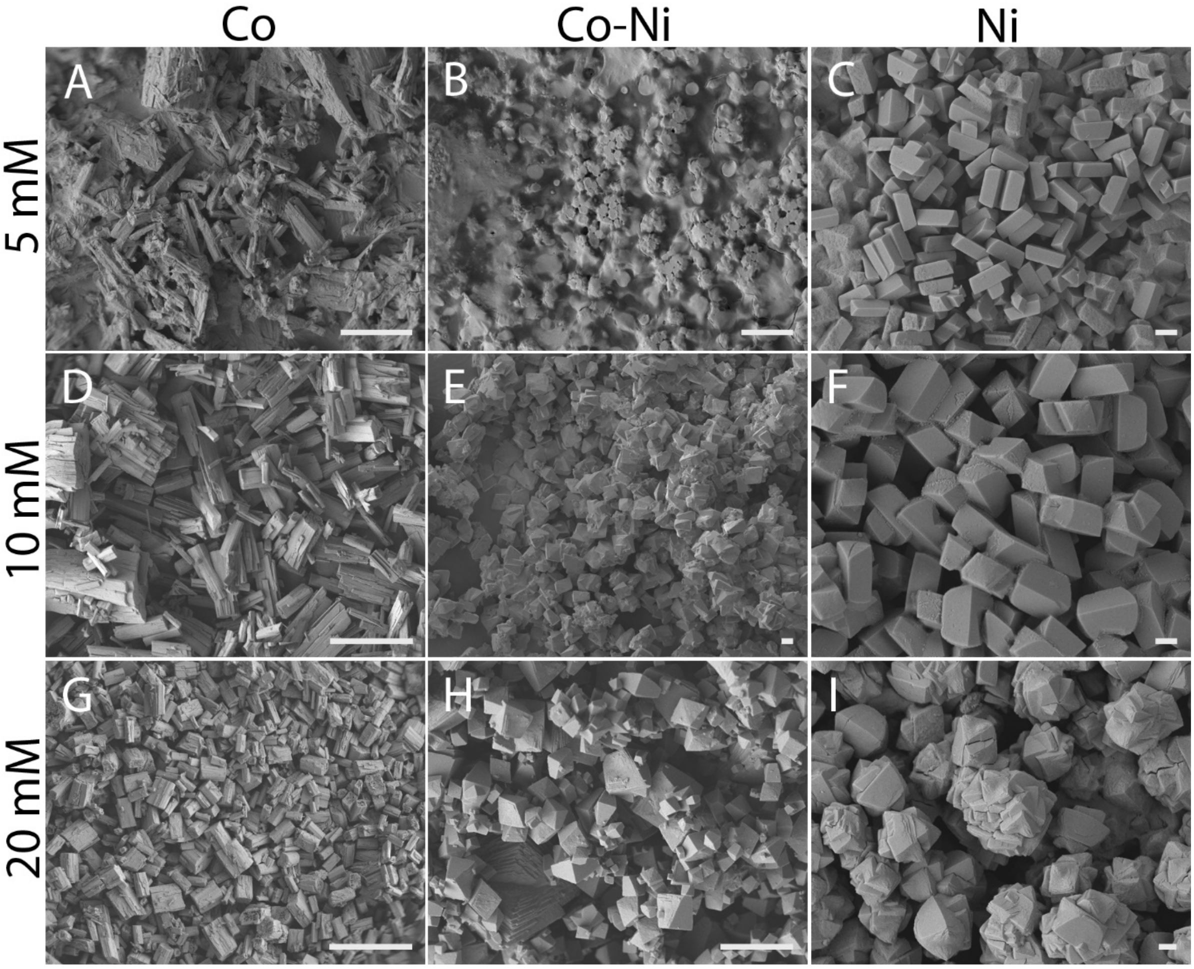

Fig. S1. Oxalate biomineral precipitate morphologies. SEM images showing Co, Co-Ni and Ni precipitate morphology following precipitation using *A. niger* synthetic struvite supernatants adjusted to pH 2.5. Images are (A, B, C) Co, Co-Ni and Ni precipitates obtained from a 5 mM initial metal concentration respectively, (D, E F) Co, Co-Ni and Ni precipitates obtained from a 10 mM initial metal concentration respectively and (G, H, I) Co, Co-Ni and Ni precipitates obtained from a 20 mM initial metal concentration respectively. Scale bars represent (A, G) 100 µm, (B, D, E, H) 10 µm and (C, F, I) 1 µm. Representative images are shown and were obtained using a JEOL SM-7400F field emission scanning electron microscope operating at an accelerating voltage of 5 keV.

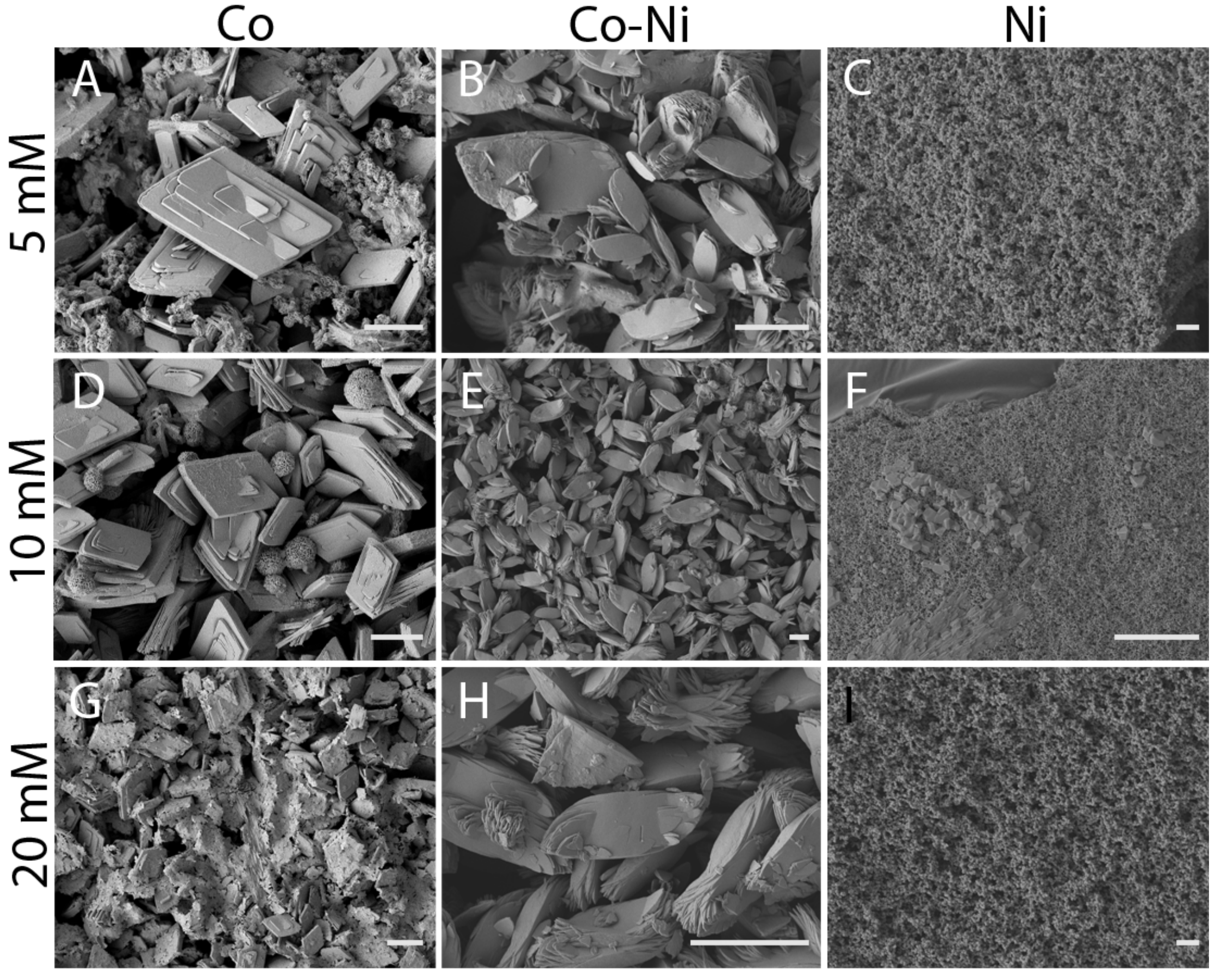

Fig. S2. Phosphate biomineral precipitate morphologies. SEM images showing Co, Co-Ni and Ni precipitate morphology following precipitation using *A. niger* struvite supernatants adjusted to pH 7.5 for phosphate precipitation following oxalate removal. Images are (A, B, C) Co, Co-Ni and Ni precipitates obtained from a 5 mM initial metal concentration respectively, (D, E F) Co, Co-Ni and Ni precipitates obtained from a 10 mM initial metal concentration respectively and (G, H, I) Co, Co-Ni and Ni precipitates obtained from a 20 mM initial metal concentration respectively. Scale bars represent 10 µm and (C, I) 1 µm. Representative images are shown and were obtained using a JEOL SM-7400F field emission scanning electron microscope operating at an accelerating voltage of 5 keV.
